# Cough-Induced Contraction Response Time and Strength of the Pelvic Floor Muscles Between Women with and Without Stress Urinary Incontinence

**DOI:** 10.3390/s25185914

**Published:** 2025-09-22

**Authors:** Elora dos Santos Silva de Lima, Erica Feio Carneiro, Karina Moyano Amorim, César Ferreira Amorim, Adriano de Oliveira Andrade, Paulo Roberto Garcia Lucareli, Daniela Aparecida Biasotto-Gonzalez, Fabiano Politti

**Affiliations:** 1 Postgraduate Program in Rehabilitation Sciences, Physical Therapy Department, Nove de Julho University, São Paulo 014019-002, Brazilericarneiro@yahoo.com.br (E.F.C.); biasottogonzalez@gmail.com (D.A.B.-G.); 2 Department of Human Movement Sciences, State University of Pará, Belém 66087-662, Brazil; 3 Postgraduate Program in Rehabilitation Sciences, Physical Therapy Department, University Cidade de São Paulo, São Paulo 03071-000, Brazil; 4 Postgraduate Program in Electrical and Biomedical Engineering, Centre for Innovation and Technology Assessment in Health, Federal University of Uberlândia (UFU), Uberlândia 38408-144, Brazil

**Keywords:** dynamometer, stress urinary incontinence, pelvic floor muscles, reflex, cough

## Abstract

Anatomic and functional changes in the pelvic floor muscles (PFMs) have been associated with stress urinary incontinence (SUI). The aim of this study is to compare cough-induced contraction response time and PFM strength in women with and without SUI. This cross-sectional study evaluated 40 women (20 with and 20 without SUI) aged 20 to 60 years. PFM strength was measured using a vaginal dynamometer. The cough signal was captured using an accelerometer, and activity of the right external oblique muscle was measured using surface electromyography. All signals were synchronized and recorded using the same signal acquisition module. Analysis of covariance (ANCOVA) with the Bonferroni post hoc test was used to compare the dynamometric data between groups (control and SUI). No significant differences were found between groups regarding variables related to PFM strength, but significant differences were found for activation time of the PFMs (F = 59.42, *p* < 0.0001, η_p_^2^ = 0.76), activation time of the external oblique muscle (F = 6.55, *p* = 0.004, η_p_^2^ = 0.26), and cough pulse time (F = 3.32, *p* = 0.04, η_p_^2^ = 0.15). Women with stress urinary have a delayed cough-induced contraction response of the pelvic floor muscles, but no difference in the contraction force of these muscles was found in comparison to women without stress urinary incontinence.

## 1. Introduction

Urinary continence in women is determined by mechanisms that involve the urethral sphincteric closure and support systems associated with the pelvic floor, such as the anterior vaginal wall, endopelvic fasciae, arcus tendineus fasciae pelvis, and pelvic floor muscles (PFMs) [1]. Functional changes in these structures have been associated with stress urinary incontinence (SUI) [2], with a prevalence of approximately 60% among women [3]. This clinical condition is characterized by the loss of a small amount of urine associated with some type of effort, such as coughing, sneezing, squatting, physical exertion, and the practice of sports involving jumping, fast running, and rotational movements [2,4].

In healthy continent women, activation of the PFMs before or during physical exertion seems to be an automatic anatomic response, so an unconscious contraction [5]. Thus, the pre-contraction, also called the fast-feed-forward loop of PFMs, is considered to be one of the main prevention mechanisms against urinary incontinence [6,7] by providing an increase in urethral pressure and consequent maintenance of a positive pressure gradient between the urethra and bladder when intra-abdominal pressure is increased during effort [8]. Coughing, for example, triggers a coordinated contraction of the thoracic, abdominal, and pelvic muscles, which increases intra-abdominal pressure, thereby leading to leakage of urine in the case of women with SUI [9,10]. Some authors have already reported that the activity of the external oblique and transversus abdominis muscles is more pronounced in women with symptoms of SUI compared to continent women [11]. This knowledge has been considered in the development of training protocols that emphasize the knack of knowing when to contract across everyday life events [12,13].

To date, however, no single theory is capable of explaining the variety of results found in investigatory exams in women with SUI [14]. The complexity of the physiopathology of this condition seems to extend beyond mechanical dysfunction, such as a possible alteration in sensory feedback in incontinent women [15]. Moreover, the cough-induced co-contraction of the PFMs is a mechanism that may be associated with SUI and should be further explored in future studies.

In continent women with normal PFM function, coughing produces timely compression of the PFMs, with additional external support to the urethra, reducing the velocity and acceleration of the PFMs and urethra [16]. As coughing is considered a provoking factor that can lead to urinary incontinence [17], this clinical condition may stem from a delay in the contraction of the PFMs in these patients.

Although it has been suggested that the PFM contraction response time in the presence of an increase in intra-abdominal pressure may be an important physiological action for the maintenance of urinary continence, [17] this relationship has not been clarified in the literature and requires a better understanding, as suggested in a previous study [18].

The aim of the present study is to compare cough-induced contraction response time and PFM strength in women with and without SUI.

## 2. Materials and Methods

### 2.1. Participants

A cross-sectional observational study was conducted with 40 women to explore variables referents cough-induced contraction response time and PFM strength in women with (n = 20) and without (n = 20) SUI, recruited from physical therapy centers at the university and locations near the university. This study received approval from the local human research ethics committee (certificate number: 1,799,546), and all participants signed a statement of informed consent.

The sample size was calculated based on a study conducted by Chamochumbi et al. [19] considering the mean and standard deviation (±) of the maximum strength of PFMs recorded with a dynamometer of women with and without SUI, the values being, respectively, 0.1 ± 0.1 N and 0.3 ± 0.2 N. For the calculation of the sample, it was considered α = 0.05 and 1-β = 0.95. A minimum of 18 individuals was determined. This calculation was performed using G*Power 3.1.9.2 software.

The inclusion criteria for women with SUI was a complaint of urinary leakage in the previous three months, a positive answer to question three of the Three Incontinence Questions used to distinguish urgency urinary incontinence and SUI in adult women [20], and pad test findings (≥2 g of leakage in 24 h) [21].

For the control group, healthy women with no complaints of urinary leakage were included.

The exclusion criteria were current pregnancy or having given birth in the previous 12 months, organ prolapse (POP-Q > Stage II) [22], infection of the urinary tract or vaginal canal, abnormal vaginal mucosa (candidiasis), a history of urogynecological surgery, a history of degenerative neurological disease or any adverse health condition that could interfere with the measurement of PFM strength, and current use of an analgesic or muscle relaxant. Women who reported “moderately” or “a lot” on the scale of symptoms related to “urgency” and “overactive bladder” on the King’s Health Questionnaire (KHQ) were also excluded from the study [23].

### 2.2. Blinding

Independent evaluators performed the following procedures. Evaluator 1: triage and evaluation of clinical characteristics; Evaluator 2: vaginal dynamometer data collection; Evaluator 3: vaginal dynamometer signal processing and statistical analysis. Evaluators 2 and 3 were blinded in relation to the groups.

### 2.3. Clinical Evaluation

The tests were performed in a single session. The clinical characteristics of women with SUI were assessed by a physiotherapist with at least 5 years of experience through the following instruments: (i) the impact of urinary continence was evaluated using the International Consultation on Incontinence Questionnaire Short Form (ICIQ-SF; 0 to 21 points; ≥3 points indicates incontinence, with higher scores denoting greater severity) [24]; (ii) severity of urinary leakage was evaluated using the Protection, Amount, Frequency, Adjustment and Body Image (PRAFAB) Questionnaire (≥14 points severe urinary incontinence) [25]; and (iii) quality of life was evaluated using the KHQ (0 to 100 points, with higher scores denoting poorer quality of life) [23].

### 2.4. Instruments

A four-channel signal acquisition system (EMG System do Brasil**^®^**, São José dos Campos, Brasil) with an analog bandpass filter from 10 and 500 Hz, common rejection mode of 120 dB, and analog/digital (A/D) converter board with 16 bits of resolution was used to capture the dynamometric, surface electromyographic (sEMG), and accelerometric signals. The sEMG signal was captured using bipolar electrodes with a 20-fold gain. The sampling frequency for all signals (EMG, dynamometer, and accelerometer) was 2000 Hz.

The vaginal dynamometer consisted of a shaft measuring 70 mm in length and 22 mm in diameter (Power Gyneco, EMG System do Brasil**^®^**, São José dos Campos, Brasil). Force was measured in Newton (N). The triaxial accelerometer had a sensitivity range of ±16 G (1 g = 9.8 m/s^2^) (ACL-3, EMG System do Brasil**^®^**, São José dos Campos, Brasil).

The data were collected using a personal computer. All equipment remained disconnected from the electrical grid during the readings to avoid interferences.

### 2.5. Procedures

**Figure 1:**
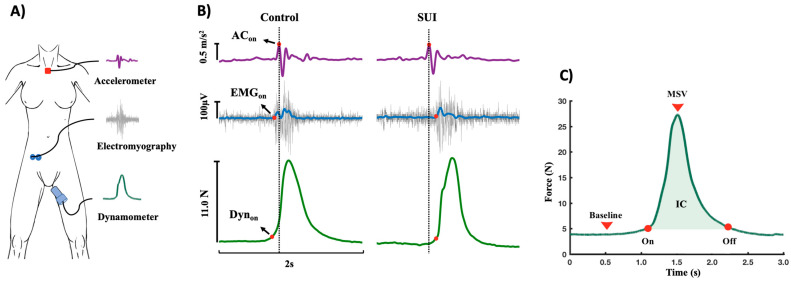
(**A**) Equipment. Accelerometer: collected the acceleration provided by cough with accelerometer positioned on suprasternal notch. Electromyography: collected the surface electromyographic activity of the right external oblique muscle of the abdomen. Dynamometer: collected the pelvic floor muscle (PFM) strength signal during cough. (**B**) Signs collected during cough. Dyn_on_: PFM onset of the vaginal dynamometer signal. AC_on_: cough onset from accelerometer signal. EMG_on_: onset of the electromyographic signal obtained from the right internal oblique. N: Newton. m/s^2^: meters per second. µV: microvolts. (**C**) Contraction of pelvic floor muscles collected using a dynamometer during cough. Baseline: passive PFM strength. Onset and offset of the cough signal. MSV: maximum strength value. CI: contraction impulse.

A physiotherapist with experience in evaluating PFMs in women with urinary incontinence conducted the tests beginning on the first day after the end of the menstrual period. After emptying the bladder, the participant was placed in the dorsal lithotomy position. The accelerometer was positioned on the suprasternal notch (Figure 1A). Disposable self-adhesive circular silver/silver chloride electrodes (Kendall Madi-Trace^®^, Cardinal Health 200, Dublin, OH, USA) measuring 10 mm in diameter (inter-electrode distance: 20 mm center to center) were placed on the right external oblique (REO), positioned obliquely approximately 45 degrees (parallel to a line connecting the most inferior point of the costal margin of the ribs and the contralateral pubic tubercle) above the anterior superior iliac spine at the level of the umbilicus (Figure 1A) [26]. This muscle was chosen for having the best amplitude of the sEMG signal due to the lower concentration of fat in comparison to the rectus abdominis muscle. A ground electrode was placed on the tibial tuberosity. Electrode placement was verified by inspection of the signal during voluntary contraction.

For the tests, the dynamometer was sanitized and covered with a condom (OLLA^®^, Hypermarcas S.A., São Paulo, Brasil). After each session, the condom was discarded, and the dynamometer was disinfected again. After insertion of the dynamometer, the participant was instructed to relax the PFMs for the recording of passive force (baseline) for a period of up to 10 s. The participant was then instructed to perform a strong cough at the end of expiration at the command of the examiner for a total of four repetitions, with a 10-s rest interval between each command. After initial training to familiarize the patient with the method, the procedure was repeated three times with a two-minute interval between readings.

The data were recorded considering the PFM contraction force on the sagittal plane. The insertion depth of the shaft of the dynamometer was not standardized, as the equipment was designed for the precise measurement of PFM contraction force independently of the location on the shaft on which the effort was performed [27]. No participants reported any discomfort either during or after the test sessions.

### 2.6. Signal Processing

The signals were processed and analyzed using routines developed in MATLAB^®^, version R2022 (The MathWorks Inc., Natick, MA, USA). All raw **s**EMG data were linearly enveloped by rectifying and low-pass filtering (4th order Butterworth filter; cutoff: 10 Hz). Movement artifacts of the signals from the dynamometer and accelerometer were removed using a 10-Hz low-pass filter. To calculate the contraction response time of the PFMs and REO, it was first necessary to determine the onset of the (EMG_on_) and dynamometer (Dyn_on_) signals. The onset was calculated from the mean of signal times three standard deviations of the baseline calculated from a 50-ms window near the onset of the contraction [28]. The offset of the cough signal obtained on the dynamometer (Dyn_off_) was determined as the point at which sEMG activity returned to the pre-movement baseline level with no additional deviation. To cough onset obtained by the accelerometer (AC_on_), the signal was first differentiated, and then the peaks were detected by using the Matlab function “findpeaks” (Figure 1B). Visual inspection was also performed to confirm the effectiveness of the automatic procedures.

The PFM and REO contraction time in relation to the cough (accelerometer signal) was calculated beginning with the onset of each signal in the following manner: PFMtime = AC_on_ − Dyn_on_; REO time = AC_on_ − EMG_on_. Positive values indicated that contraction of the muscles preceded the cough, and negative values indicated that contraction occurred after the cough.

The PFM contraction force provided by the cough was evaluated based on the dynamometer signal using variables described by Nagano et al. [27].

(i) Passive PFM strength: The mean value of the baseline (passive force) considering the 5s that preceded the beginning of the PFM contraction.
(ii) Maximum strength value (MSV) during cough: Calculated as the peak force value obtained during the effort minus the baseline value recorded just before the beginning of the PFM contraction
(iii) PMF contraction impulse (CI) was calculated considering:
  $\mathrm{IC}=\int \begin{array}{c} t_{f}\\ t_{i} \end{array}\;$F*dt*, in which F is force during the contraction (N). Based on this definition, the impulse of the contraction corresponds to the area beneath the maximal force-time curve, with $t_{i}$ representing the initial contraction time and $t_{f}$ denoting the moment when the maximal contraction force is achieved.
(iv) Average contraction force (AF) of the PFMs. Calculating the area under the force/time curve, that is, the impulse (IC), and because the force imparting an impulse can generally vary in time, it is convenient to define a time average contraction force (AF) as:
  $\mathrm{AF}=\frac{1}{\Delta t}\int \begin{array}{c} t_{f}\\ t_{i} \end{array}\;$*Fdt* in which F is force (N) or $\mathrm{AF}=\frac{\mathrm{IC}}{\Delta t}$, in which $\Delta t=t_{f}-t_{i}$.
(v) Cough pulse duration time was also calculated: CPT = Offset − Onset (Figure 1C).

### 2.7. Statistical Analysis

The reproducibility of the data regarding the onset and offset time and the dynamometric variables of the PFMs recorded during cough in each group (Control and SUI) was estimated through test/retest analyses, considering three trials per subject. Intra-class correlation coefficients (ICC) were interpreted using the following criteria: 0.00–0.39 = poor; 0.40–0.59 = fair; 0.60–0.74 = good; and 0.75–1.00 = excellent [28].

The Shapiro–Wilk test was used to determine the normality of the data distribution. Demographic data from the two groups and intra-group variables were compared using either the independent *t*-test or the Mann–Whitney test, depending on the distribution of the data. For the analysis of variables related to contraction response time and PFM strength, the average of twelve repetitions (four repetitions per session across three testing sessions) was used. Analysis of covariance (ANCOVA) was used to compare variables between groups, with age included as a covariate. Age was included as a covariate in these analyses because this variable was significantly different between the groups (*p* < 0.05). The Bonferroni post-hoc test was applied when ANCOVA indicated significant differences between the groups. A *p*-value < 0.05 was considered indicative of statistical significance (demographic data, ANCOVA, and Bonferroni post-hoc test).

The partial eta squared value (η_p_^2^) was used to calculate the effect size of the interactions, the results of which were interpreted based on Cohen: <0.01 = small effect; 0.06 = moderate effect; and ≥0.14 = large effect. All statistical analyses were performed with the aid of SPSS version 20.0 (IBM Corporation, Armonk, NY, USA). 

## 3. Results

### 3.1. Sociodemographic Characteristics

Table 1 displays the demographic and clinical data of the 40 participant women (20 control and 20 SUI), and significant differences were found with regard to age (*p* = 0.02). The table also displays the scores of the KHQ (quality of life), ICIQ-SF (impact of urinary incontinence), and PRAFAB (severity of urinary leakage) questionnaires in the group with SUI.

### 3.2. Intra-Session Reliability of Contraction Response Time and Strength of the PFMs

Table 2 displays the estimated ICCs for the onset and offset data and dynamometric variables of the PFMs recorded during cough considering three trials per subject. Excellent reproducibility was demonstrated (ICC: 0.76 to 0.98).

### 3.3. PFM Function

Table 3 displays the variables related to the contraction response time and strength of the PFMs during cough. In the inter-group analysis, significant differences in PFMtime (*p* < 0.001), REOtime (*p* = 0.001), and CPT (*p* = 0.04) were found. However, no significant differences were found with regard to variables related to the contraction force of the PFMs (Baseline, MSV, IC, AF) (*p* > 0.05).

As age differed significantly between groups, it was considered co-variable for the comparison of the data. ANCOVA confirmed the significant difference between groups for the response time variables: PFMtime (*p* < 0.0001; η_p_^2^ = 0.76), REOtime (*p* = 0.004; η_p_^2^ = 0.26), and CPT (*p* = 0.04; η_p_^2^ = 0.15) (Table 3).

## 4. Discussion

The present findings of this exploratory study demonstrated that women with SUI have a delayed cough-induced PFM contraction response in comparison to women without SUI. In contrast, no differences between groups were found with regard to the intensity of the PFM contraction force. The excellent reliability of the variables used in this study indicates that the contraction response time and strength of the PFMs can be used as reliable measures (Table 2).

The possible change in the components of muscle strength in women with SUI was not confirmed by the results in the present study, as no significant differences between groups were found for any of the variables (Baseline, MSV, IC, and AF) related to the contraction force of the PFMs (Table 3). Previous studies have demonstrated that the development of active strength in the tissues of the pelvic floor may [19] or may not [29] be significantly reduced in women with SUI. The divergent findings in the literature regarding PFM contraction force may stem from differences in the equipment used to measure PFM strength. The dynamometer used in the present study was designed with a load cell positioned in the center of the shaft so that PFM contraction force could be measured precisely, independently of the point on the shaft at which the effort was exerted [27]. Similar care was taken in the construction of the equipment used in a study that also found no difference in PFM contraction force between continent women and women with SUI [30]. In that study, the strain gauges were mounted in a Wheatstone bridge using a differential configuration, allowing only the voltage difference between two opposing pairs of strain gauges to be measured. The study that reported a significant difference in PFM contraction force between these two populations [19] did not provide details about the construction characteristics of the vaginal dynamometer used.

Although the results of this study should be interpreted with caution given its exploratory nature, the findings suggest that urinary incontinence in women with SUI while coughing seems not to be due to weakness of the PFMs but possibly due to a delay in the contraction or motor coordination of these muscles. This possibility has been raised in previous studies, in which SUI was associated with the possibility of fascial support defects and/or altered PFM and abdominal muscle coordination rather than a change in the contraction force of the PFMs [31]. Although the physiology of SUI is complex and the respective active and passive mechanisms of the structures that compose the pelvic floor remain little known, the altered PFM activation time during cough in the present study strengthens the previously described argument regarding the possible existence of specific neuromuscular disorders that affect women with SUI [7].

In general, the maintenance of continence in the occurrence of a load with a high, fast impact on the PFMs can only be ensured by a rapid muscle co-contraction [7]. A previous study demonstrated that the PFMs contract in a modulated manner during successive coughing efforts [6]. However, the gradual adaptation of these muscles may be one of the main factors that contribute to continence, as this modulated response is absent in individuals with urinary or fecal incontinence [6].

The delayed contraction of the REO muscle in relation to the contraction of the PFMs in the control group (Table 3) indicates that the activity of the PFMs occurs prior to the increase in pressure in the abdomen, as described elsewhere [8]. This demonstrates that the PFM response in healthy women can be pre-programmed. In women with SUI, however, this pattern appears to be altered, as demonstrated by the finding that the onset of REO muscle and PFM activity was practically the same (Table 3). The delayed contraction response in both the REO muscle and PFMs in the women with SUI and the similarities between groups regarding the strength variables (Baseline, MSV, CI, and AF) suggest that PFM response time plays a significant role in the urinary continence mechanism, as reported in a previous study [32]. Therefore, while impaired modulation of the PFMs’ response to stress could represent a key pathophysiological factor contributing to SUI, this observation warrants further investigation and should be validated or refuted in future studies.

PFM contraction during a cough must be pre-programmed by the central nervous system in preparation for the increase in intra-abdominal pressure in continent women, but this pre-programming is described as altered in women with SUI [6,33]. Thus, there is strong evidence that the PFM contraction prior to a voluntary cough is not a simple spinal reflex but the result of the involvement of higher and/or more complex integrative centers involving complex, coordinated neural activity, which implies the participation of the brain and autonomic neural pathways [33]. All these observations may be an indication that the efficiency of the PFMs is dependent not only on the anatomic integrity of the pelvic floor and the strength of these muscles but also on the response of the central nervous system, in which coordinated responses must be generated so that the activities of different muscle occur at the correct moment and with the appropriate level of strength for the maintenance of urinary continence.

PFM strength training programs are reported to favor better urinary continence [34]. This positive result is attributed to both neurological and morphophysiological changes found after PFM strength training, such as increased muscle strength, hypertrophy (whole-muscle growth), and the enhanced automatic function of PFM contractions [35,36]. Thus, the delayed contraction of PFMs observed in this study lends support to the previously reported finding that PFM strength training programs can also improve the response time of these muscles. However, given the exploratory nature of this study, this interpretation should be approached with caution, and further research is needed to confirm whether PFM training directly influences contraction timing in women with SUI.

Thus, evaluating the PFM contraction response time before and after a physiotherapeutic program in clinical trials that compare PFM strength and co-contraction training protocols may lead to a better understanding of the mechanisms involved in this dysfunction.

The present findings can be considered another step toward the understanding of female SUI. The fact that the intensity of the cough was not controlled during the data collection procedure should not be considered a possible source of bias due to the fact that the strength variables were similar between the two groups. Moreover, all variables used to determine the contraction response time of the PFMs exhibited excellent reliability in both groups. The use of age as co-variable in the statistical analysis is another factor that diminishes the risk of bias in this study.

### Limitations

A possible limitation of this study may have been the lack of urodynamic evaluation in patients with SUI since urodynamic investigation is a functional assessment of the lower urinary tract to provide objective pathophysiological explanations for symptoms and/or dysfunction of the lower and upper urinary tracts. Therefore, the lack of this evaluation limited the understanding of the delay in the contraction response of the PFMs found in this study. This is a limitation that must be overcome in future studies. A second limitation of the study is that the dynamometer does not precisely differentiate intra-abdominal pressure from the pressure exerted by the PFMs. Thus, it is not possible to affirm whether or not the PFM contraction data obtained with the dynamometer were influenced by abdominal pressure.

The third limitation is the sample size. The number of individuals may not precisely represent the entire population with SUI. Thus, the results of this study should be analyzed with caution.

Factors such as muscle coordination, endurance, and quality are often considered in therapeutic approaches used as treatment for SUI [37,38] and should, therefore, be taken into consideration for a comprehensive assessment of PFM function. Recent studies have demonstrated that some additional factors may also contribute to the development of SUI, such as excess weight, obesity [39], testosterone levels [40], and lifestyle habits [41].

## 5. Clinical Implications

Although the results of this study should be interpreted with caution due to the methodological limitations previously mentioned, the delayed contraction response of the PFMs induced by coughing may have important implications for rehabilitation protocols in women with SUI. Effective PFM training depends on the precise timing of muscle activation to ensure adequate support for the bladder and urethra during activities that elevate intra-abdominal pressure, such as coughing, sneezing, or lifting [42,43]. A delayed contraction response may reflect neuromuscular dysfunction, potentially resulting in inadequate support and persistent urinary leakage.

This finding suggests that rehabilitation protocols should be adapted to address the timing of muscle activation, not just strength and endurance. Interventions may need to incorporate biofeedback, neuromuscular re-education, and external cues to improve the speed and coordination of pelvic floor contractions. Additionally, therapists may need to emphasize anticipatory contractions—training patients to engage their PFMs before stress-inducing activities occur. By refining these protocols, clinicians can enhance treatment outcomes and improve the quality of life for women with SUI.

## 6. Conclusions

In the present study, women with stress urinary incontinence presented a delay in the contraction response of the pelvic floor muscles induced by cough. In contrast, no changes were found in variables related to the intensity of the contraction force of these muscles (contraction impulse, passive strength, mean strength, and maximum strength) in this group of patients.

## Figures and Tables

**Table 1:** Mean and standard deviation of demographic and clinical variables of women with stress urinary incontinence (SUI) and healthy controls.

	Control (n = 20)	SUI (n = 20)	*p*-Value
Age (years)	35.40 ± 8.15	44.15 ± 6.83	0.02 *
Body mass index (Kg/m^2^)	27.87 ± 3.97	27.72 ± 3.68	0.68
Parity	1.80 ± 0.89	2.15 ± 1.08	0.26 ^†^
PRAFAB		13.12 ± 2.89	
ICIQ-SF		12.18 ± 4.22	
Pad test 24-h (g)		14.41 ± 19.61	
KHQ scores			
General health perceptions		34.28 ± 20.19	
Impact of incontinence		50.15 ± 28.31	
Role limitations		43.45 ± 29.94	
Physical limitations		48.01 ± 34.68	
Social limitations		19.45 ± 20.81	
Personal relationships		18.64 ± 20.85	
Emotions		36.62 ± 30.01	
Sleep/Energy		32.19 ± 29.25	
Severity measures		49.45 ± 26.64	

g: grams; ICIQ-SF: International Consultation on Incontinence Questionnaire Short Form; KHQ: King’s Health Questionnaire; Parity: number of births; PRAFAB: Protection, Amount, Frequency, Adjustment, and Body Image Questionnaire; * Statistically significant difference (*p* < 0.05, independent *t*-test); ^†^ Mann–Whitney test.

**Table 2:** Reliability of different time indices and force variables of pelvic floor muscles.

	Control	SUI
	ICC (95% CI)	ICC (95% CI)
**Time analysis (s)**		
PFMtime	0.81 (0.65 to 0.91)	0.79 (0.62 to 0.90)
REOtime	0.85 (0.72 to 0.93)	0.96 (0.96 to 0.98)
CPT	0.95 (0.90 to 0.98)	0.96 (0.96 to 0.98)
**Force analysis (N)**		
Baseline	0.88 (0.77 to 0.95)	0.76 (0.57 to 0.89)
MSV	0.96 (0.96 to 0.98)	0.94 (0.88 to 0.97)
IC	0.98 (0.96 to 0.99)	0.97 (0.94 to 0.99)
AF	0.96 (0.96 to 0.98)	0.87 (0.75 to 0.94)

ICC: intraclass correlation coefficient; 95%CI: 95% confidence interval of the mean; PFMtime: contraction time of pelvic floor muscles in relation to cough; REOtime: contraction time of right external oblique muscle in relation to cough; CPT: cough pulse time; Baseline: passive force of pelvic muscle floor; MSV: maximum pelvic muscle floor strength value; CI: contraction impulse of pelvic floor muscles; AF: average contraction force of pelvic floor muscles.

**Table 3:** Mean and standard deviation of onset and offset time and different dynamometric variables of pelvic floor muscles recorded during cough in women with stress urinary incontinence (SUI) and healthy controls.

	Control	SUI	Mean Difference	*p*-Value
	(n = 20)	(n = 20)	(95% CI)	
**Time analysis (s)**				
PFMtime	0.11 ± 0.04	−0.14 ± 0.09 ^†^	−0.26 (−0.31 to −0.21)	<0.001 *
REOtime	0.04 ± 0.14	−0.10 ± 0.11 ^†^	−0.15(−0.24 to −0.05)	0.001 *
CPT	1.40 ± 0.30	1.15 ± 0.28	−0.24 (−0.46 to −0.02)	0.04 *
**Force analysis (N)**				
Baseline	5.19 ± 1.12	5.94 ± 2.26	0.75 (−0.49 to 2.00)	0.27
MSV	8.24 ± 2.51	9.77 ± 3.28	1.53 (−0.54 to 3.62)	0.29
IC	11.92 ± 3.63	12.18 ± 4.56	0.26 (−2.73 to 3.26)	0.67
AF	9.07 ± 1.79	9.56 ± 2.74	0.49 (−2.73 to 2.17)	0.13

PFMtime: contraction time of pelvic floor muscles in relation to cough; REOtime: contraction time of right external oblique muscle in relation to cough; CPT: cough pulse time; Baseline: passive force of pelvic muscle floor; MSV: maximum pelvic muscle floor strength value; CI: contraction impulse of pelvic floor muscles; AF: average contraction force of pelvic floor muscles; 95%CI: 95% confidence interval. * Significant difference between groups (Bonferroni post-hoc test: *p* < 0.05). ^†^ Negative values (−) signify delay in muscle contraction in relation to cough.

## Data Availability

The data analyzed during the current study are available upon reasonable request from the corresponding author.
